# Risk Factors Associated with Colorectal Cancer in a Subset of Patients with Mutations in *MLH1* and *MSH2* in Taiwan Fulfilling the Amsterdam II Criteria for Lynch Syndrome

**DOI:** 10.1371/journal.pone.0130018

**Published:** 2015-06-08

**Authors:** Abram Bunya Kamiza, Ling-Ling Hsieh, Reiping Tang, Huei-Tzu Chien, Chih-Hsiung Lai, Li-Ling Chiu, Tsai-Ping Lo, Kuan-Yi Hung, Chun-Yi Wang, Jeng-Fu You, Chao A. Hsiung, Chih-Ching Yeh

**Affiliations:** 1 School of Public Health, College of Public Health and Nutrition, Taipei Medical University, Taipei, Taiwan; 2 Department of Public Health, College of Medicine, Chang Gung University, Tao-Yuan, Taiwan; 3 Colorectal Section, Chang Gung Memorial Hospital, Tao-Yuan, Taiwan; 4 Department of Nutrition and Health Sciences, Chang Gung University of Science and Technology, Tao-Yuan, Taiwan; 5 Institute of Population Health Sciences, National Health Research Institutes, Miaoli, Taiwan; 6 Graduate Institute of Biomedical Sciences, Chang Gung University, Tao-Yuan, Taiwan; 7 Department of Public Health, China Medical University, Taichung, Taiwan; University of Texas MD Anderson Cancer Center, UNITED STATES

## Abstract

**Background and Aim:**

Lynch syndrome, caused by germline mutations in mismatch repair genes, is a predisposing factor for colorectal cancer (CRC). This retrospective cohort study investigated the risk factors associated with the development of CRC in patients with *MLH1* and *MSH2* germline mutations.

**Methods:**

In total, 301 *MLH1* and *MSH2* germline mutation carriers were identified from the Amsterdam criteria family registry provided by the Taiwan Hereditary Nonpolyposis Colorectal Cancer Consortium. A Cox proportional hazard model was used to calculate the hazard ratios (HRs) and 95% confidence intervals (CIs) to determine the association between the risk factors and CRC development. A robust sandwich covariance estimation model was used to evaluate family dependence.

**Results:**

Among the total cohort, subjects of the Hakka ethnicity exhibited an increased CRC risk (HR = 1.62, 95% CI = 1.09–2.34); however, those who performed regular physical activity exhibited a decreased CRC risk (HR = 0.62, 95% CI = 0.41–0.88). The CRC risk was enhanced in *MLH1* germline mutation carriers, with corresponding HRs of 1.72 (95% CI = 1.16–2.55) and 0.54 (95% CI = 0.34–0.83) among subjects of the Hakka ethnicity and those who performed regular physical activity, respectively. In addition, the total cohort with a manual occupation had a 1.56 times higher CRC risk (95% CI = 1.07–2.27) than did that with a skilled occupation. Moreover, *MSH2* germline mutation carriers with blood group type B exhibited an increased risk of CRC development (HR = 2.64, 95% CI = 1.06–6.58) compared with those with blood group type O.

**Conclusion:**

The present study revealed that Hakka ethnicity, manual occupation, and blood group type B were associated with an increased CRC risk, whereas regular physical activity was associated with a decreased CRC risk in *MLH1* and *MSH2* germline mutation carriers.

## Introduction

Lynch syndrome is a hereditary disorder caused by a mutation in mismatch repair (MMR) genes, and patients affected by this syndrome are at a higher risk of developing colorectal cancer (CRC) early in life [1–3]. In Southeastern Asia, Taiwan has the second highest incidence of CRC after Japan; moreover, this incidence is higher in men (42/100,000) than in women (31/100,000) [4]. In addition, CRC is the second and third leading cause of cancer death in Taiwanese women and men, respectively [5]. The cumulative prevalence of *MLH1*, *MSH2*, and *MSH6* germline mutations in Lynch syndrome is approximately 5.6%, and 3% of all CRCs are attributed to Lynch syndrome [6,7]. According to the NCBI database, *MLH1* and *MSH2* germline mutations contribute to approximately 90% of all mutations associated with Lynch syndrome; *MSH6* contributes to 7%–10%, and *PMS2* contributes to less than 5% of these alterations [8]. In addition, deletions in the *EPCAM* gene have also been reported in approximately 1%–3% of Lynch syndrome patients within Dutch and German populations [9].

Several epidemiological studies have consistently demonstrated that modifiable lifestyle and dietary factors, such as smoking, alcohol consumption, meat intake, and increased body mass index, increase the CRC risk in patients with Lynch syndrome [10–13]. However, these studies have been performed only in Western populations. Moreover, Asian and Western populations differ in culture, lifestyle factors, and dietary intake patterns [14]. Identifying risk factors associated with CRC in Lynch syndrome patients is crucial because they are at a higher risk of developing CRC in early life.

To date, only one case-control study has investigated this association in Taiwan and reported that decreased physical activity, low or moderate coffee consumption, cigarette smoking, and alcohol intake were associated with CRC; however, this case-control study excluded Lynch syndrome carriers [15]. Another case-control study examining a multiethnic study population reported a significant association between regular physical activity (≥1 hour/week) and a lower risk of colon polyps and adenomas [16]. Studies have consistently shown that smoking, alcohol consumption, and meat intake increase the risk of CRC development [10–12]. Other studies have reported that regular physical activity and vegetable and fruit consumption are associated with a decreased CRC risk [15–18]. Similarly, most studies investigating risk factors associated with CRC development have focused only on sporadic CRC or asymptomatic cases [15,19].

This retrospective cohort study investigated possible risk factors associated with CRC development in patients with *MLH1* and *MSH2* germline mutations. To our knowledge, no study has investigated this association in patients with Lynch syndrome in the Chinese population.

## Material and Methods

### Participant recruitment

The Amsterdam II criteria were adopted for patient enrollment in the Amsterdam criteria family registry provided by the Hereditary Nonpolyposis Colorectal Cancer Consortium of the National Health Research Institutes in Taiwan. Clinical data, including that on histological characteristics and molecular genetic analysis, were collected from all index patients from seven hospitals and medical centers around Taiwan from May 2002 onwards. The search for germline mutations in the *MLH1* and *MSH2* genes was performed in each index patient. The probands were requested to contact their relatives to seek permission for their enrollment in the registry; details of the participants were described previously [20].

Written informed consent was obtained from all study participants, and the study protocol was approved by the Institutional Review Board of Taipei Medical University and the National Health Research Institutes. As of February 2012, 135 Amsterdam II criteria families, comprising 1,014 family members, had been registered and their genetic analyses completed. Pathogenic mutations in the *MLH1* or *MSH2* gene were identified in 82 of 135 families (60.7%). Information of the mutated genes, germline mutations, and family ID is shown in S1 Table. We studied 303 carriers harboring a germline mutation in one of the two MMR genes, *MLH1* or *MSH2*. Because two patients had both mutations in *MLH1* and *MSH2* genes, the total number of studied carriers was 301. One of the patients had both c.1846_1848delAAG and c.2595_2597delCAT mutations in *MLH1* and *MSH2*, respectively, and another had both c.793C>T and c.2516A>G mutations in *MLH1* and *MSH2*, respectively.

### Data collection

Professional nurses in the colorectal surgery department were trained for interviewing all probands and their relatives at the hospitals. Nurses had no prior knowledge of the study hypotheses regarding diet, lifestyle, and CRC, and all interviews were administered uniformly in the wards. After obtaining informed consent, a standardized interview was conducted using a structured questionnaire covering questions on sociodemographic characteristics, lifestyle factors (physical activities, cigarette smoking, alcohol intake, and tea and coffee intake), dietary factors, and medical history. The reliability (standardized Cronbach’s alpha) of this questionnaire was 0.92, based on the lifestyle and dietary variables [15].

Information on the usual dietary intake of 14 food items by the participants was obtained for 5 years preceding the date of study registry. The intake frequency was categorized into six levels: never, less than once a month, one to three times a month, once a week, two to three times a week, and almost daily. Physical activity in the last year was assessed on the basis of vigorous leisure time activities, including jogging 16 km/week, swimming more than 3.2 km/week, and participation in racket sports, martial arts, and other sports more than 5 hours/week [21]. Subjects practicing any one of these activities were defined as “Yes,” and others as “No.” Cigarette smoking and alcohol, tea, and coffee consumption were categorized into never, former, and current, based on the status of use of the participants. Because few of the subjects were former cigarette smokers or alcohol, tea, or coffee consumers (6.9%, 3%, 0.66%, and 1.6%, respectively), we combined the “former” and “current” categories to form the “ever” category. The participants were biennially followed up from May 2002 to February 2012 to obtain updates about their morbidity statuses. Reports of cancer diagnoses and the age at diagnosis were confirmed, where possible, on the basis of pathology reports, medical records, cancer registry reports, and death certificates.

### Germline mutation screening

DNA was extracted from white blood cells by following standard procedures involving sodium dodecyl sulfate proteinase K–RNase digestion and phenol–chloroform extraction. *MLH1* and *MSH2* germline mutations were tested in all probands who had a colorectal tumor exhibiting evidence of impaired MMR function as indicated by microsatellite instability or a lack of MMR protein expression in immunohistochemical analysis. Mutations were tested by performing denaturing high-pressure liquid chromatography (WAVE DNA Fragment Analysis System, Omaha, NE, USA), followed by confirmatory DNA sequencing. Large insertion and deletion mutations were detected using the multiplex ligation-dependent probe amplification (MLPA) analysis by using SALSA MLPA kit P003 according to the manufacturer’s instructions (MRC-Holland, Amsterdam, Netherlands). All participants who donated blood samples and who were related to the probands with a pathogenic mutation were tested for the same mutation identified in the proband.

### Immunohistochemistry

Immunoperoxidase staining was performed on formalin-fixed tissues. Sections (5 μm) from representative tumor blocks were deparaffinized in xylene and absolute alcohol, retrieved with heat, and treated with 3% hydrogen peroxide to eliminate endogenous peroxidase activity. Immunohistochemical staining was performed using specific mouse monoclonal antibodies for MLH1 (clone ES05, 1:100; Novocastra, Newcastle Upon Tyne, UK) and MSH2 (clone 25D12, 1:80; Novocastra), respectively, and the NovoLink Polymer Detection System (Novocastra). Slides were then counterstained with hematoxylin, mounted with Permount (Fisher Chemical, Pittsburgh, PA, USA), and examined for the extent and intensity of nuclear and cytoplasmic staining in tumor cells and for background staining in the stroma. For negative controls, these primary antibodies were replaced with phosphate-buffered saline.

### Microsatellite instability analysis

Microsatellite analysis was performed as described previously [22]. The primer panel comprised the reference panel of markers (BAT25, BAT26, D2S123, D5S346, and D17S250). Tumors with a high frequency of microsatellite instability were defined as tumors having instability in two or more markers from the reference panel [22].

### Statistical analysis

Food items were categorized into five major groups: staple, meat, vegetable, seafood, and fruit. The consumption of each food item was scored from 1 for “ate almost daily” to 6 for “never ate.” A group score was the sum of scores for the intake frequency of the individual food items. These dietary factors were tertile stratified into “low,” “medium,” and “high,” and the lowest tertile of the intake of each food item was set as a reference category.

Descriptive statistics was used to describe the sociodemographic characteristics, lifestyle factors, dietary factors, family history of cancer, and medical history. The risk was considered to begin at birth and end at the diagnosis of CRC, death, or loss to follow-up. Carriers who were not diagnosed for CRC, did not die, and were not lost to follow-up were censored at the date of their last known contact or in February 2012.

Pearson’s chi-square test was used to compare the intergroup distributions of the clinical characteristics. A Cox proportional hazard model was used to assess the hazard ratios (HRs) and 95% confidence intervals (CIs) for the association between risk factors and CRC development. Mutated MMR genes (*MLH1* or *MSH2*) and year of birth (<1940, 1940–1949, 1950–1959, 1960–1969, 1970–1979, and >1980) were adjusted for as potential confounding factors. A robust sandwich covariance estimation model was used to adjust for within-cluster (data were not independent within the groups, but independent among the groups) and within-family correlations of the age of onset [23].

The following variables were regarded as potential risk factors associated with CRC in patients with Lynch syndrome: sex, education, ethnicity, occupation, blood group, physical activity, cigarette smoking, alcohol drinking, tea consumption, coffee consumption, and intake of meat, vegetables, fruits, seafood, and staple foods. A stepwise selection procedure with default selection criteria was applied to determine whether covariates were included in the multivariate models, beginning with all potential risk factors. Moreover, a stratified analysis of mutations in MMR genes, namely *MLH1* and *MSH2*, was performed. A P value of <0.05 was considered statistically significant, and all analyses were performed using the Statistical Analysis Software (SAS) package (Version 9.4 for Windows; SAS Institute, Inc., Cary, NC, USA). All statistical tests were two-sided.

## Results

The study population comprised 209 *MLH1* and 92 *MSH2* germline mutation carriers from 75 families, accounting for a total person-time of 12,529 years. During the follow-up period, 147 (48.8%) carriers developed histologically confirmed CRC, and 109 (74.1%) were diagnosed with adenocarcinoma. Of these carriers, 145 (48.2%) were men; moreover, approximately 33.2% of the carriers attended school up to the elementary level, 35.2% attended school up to the high school level, and 31.6% attended school up to the college level (Table 1). In addition, 78.1% of the carriers were Taiwanese, 19.6% were Hakka, and the remaining 2.3% were mainland Chinese or aborigines. The median age for CRC diagnosis was 42 and 35.5 years for *MLH1* and *MSH2* germline mutation carriers, respectively. However, the proportion of *MLH1* germline mutation carriers who were Hakka and developed CRC was higher than that of *MSH2* germline mutation carriers.

**Table 1:** Sociodemographic characteristics of carriers with germline mutations in mismatch repair genes.

Variable	Total cohort (n = 301)	*MLH1*germline mutation carriers (n = 209)	*MSH2* germline mutation carriers (n = 92)
Age at diagnosis, median (IQR)^a^	41 (31–50)	42 (33–52)	35.5 (26–46)
Sex, n (%)			
Female	156 (51.8)	107 (51.2)	49 (53.3)
Male	145 (48.2)	102 (48.8)	43 (46.7)
Education level, n (%)			
Elementary	100 (33.2)	74 (35.4)	26 (28.3)
High school	106 (35.2)	69 (33.0)	37 (40.2)
College	95 (31.6)	66 (31.6)	29 (31.5)
Ethnicity, n (%)			
Taiwanese	235 (78.1)	154 (73.7)	81 (88.0)
Hakka	59 (19.6)	48 (23.0)	11 (12.0)
Others ^b^	7 (2.3)	7 (3.3)	–
Colorectal cancer, n (%)			
No	154 (51.2)	95 (45.5)	59 (64.1)
Yes	147 (48.8)	114 (54.5)	33 (35.9)
Histological diagnosis, n (%)			
Adenocarcinoma	109 (74.1)	83 (72.8)	26 (78.8)
Mucinous adenocarcinoma	15 (10.2)	11 (9.7)	4 (12.1)
Adenoma	2 (1.4)	2 (1.7)	–
Signet ring cell adenocarcinoma	1 (0.7)	1 (0.9)	–
Unknown	20 (13.6)	17 (14.9)	3 (9.1)

IQR: Interquartile range

^a^ IQR (25th–75th percentiles)

^b^ Mainland Chinese and aborigines

Table 2 shows the association of the risk factors with CRC development. The MMR gene germline mutation carriers of Hakka ethnicity exhibited a 1.65 times higher CRC risk (95% CI = 1.12–2.43) than did their Taiwanese counterparts. In addition, the carriers with a manual occupation exhibited a 1.75 times higher CRC risk (95% CI = 1.20–2.55) than did those with a skilled occupation. Furthermore, regular physical activity, ever tea consumption and high-level fruit intake were associated with a decreased CRC risk (HR = 0.58, 95% CI = 0.40–0.86; HR = 0.68, 95% CI = 0.48–0.96; and HR = 0.60, 95% CI = 0.38–0.94, respectively).

**Table 2:** Hazard ratios for association between demographic variables, lifestyle factors, or dietary factors and colorectal cancer (CRC) risk in carriers with germline mutations in mismatch repair genes.

Variable	Number of carriers	Person-Year	CRC cases	HR (95% CI) ^c^	P value
Sex					
Female	156	6,734	76	1.00	
Male	145	5,795	71	1.14 (0.83–1.58)	0.421
Education					
Elementary	100	5,235	74	1.00	
High school	106	4,112	47	0.81 (0.52–1.25)	0.348
College	95	3,182	26	0.65 (0.38–1.11)	0.113
Ethnicity					
Taiwanese	235	9,763	107	1.00	
Hakka	59	2,481	35	**1.65 (1.12–2.43)**	**0.011**
Other ^a^	7	284	5	1.66 (0.76–3.58)	0.199
Occupation					
Skilled	119	4,974	52	1.00	
Manual	112	4,973	69	**1.75 (1.20–2.55)**	**0.003**
Other ^b^	67	2,423	23	1.34 (0.76–2.35)	0.301
Blood group					
O	127	5,530	62	1.00	
A	96	3,927	50	1.06 (0.72–1.57)	0.747
B	54	2,274	28	1.21 (0.80–1.81)	0.370
AB	8	366	5	1.10 (0.44–2.76)	0.826
Regular physical activity					
No	197	8,147	109	1.00	
Yes	104	4,382	38	**0.58 (0.40–0.86)**	**0.006**
Cigarette smoking					
Never	210	8,763	102	1.00	
Ever	91	3,766	45	1.06 (0.73–1.52)	0.753
Alcohol drinking					
Never	202	8,665	105	1.00	
Ever	99	3,864	42	0.92 (0.62–1.36)	0.686
Tea consumption					
Never	120	5,412	74	1.00	
Ever	181	7,117	73	**0.68 (0.48–0.96)**	**0.023**
Coffee consumption					
Never	201	8,844	109	1.00	
Ever	100	3,684	38	0.96 (0.65–1.41)	0.826
Meat intake					
Low	107	4,022	44	1.00	
Medium	102	4,289	53	1.10 (0.75–1.62)	0.626
High	92	4,218	50	0.99 (0.65–1.52)	0.989
Vegetable intake					
Low	109	4,667	55	1.00	
Medium	101	4,098	49	1.33 (0.89–1.99)	0.162
High	91	3,764	43	0.93 (0.63–1.73)	0.717
Fruit intake					
Low	111	4,507	56	1.00	
Medium	91	4,068	56	0.94 (0.63–1.40)	0.774
High	99	3,953	35	**0.60 (0.38–0.94)**	**0.026**
Seafood intake					
Low	143	6,074	75	1.00	
Medium	115	4,574	50	0.80 (0.55–1.16)	0.241
High	43	1,880	22	0.93 (0.57–1.53)	0.789
Staple food intake					
Low	102	3,904	41	1.00	
Medium	114	4,979	65	1.16 (0.77–1.74)	0.472
High	85	3,646	41	0.86 (0.55–1.33)	0.501

^a^ Mainland Chinese and aborigines

^b^ Retirees and housekeepers

^c^ Robust variance estimation for familial correlation between risk factors and CRC risk and further adjusted for the specific MMR genes mutated in the carriers

We further investigated the association between risk factors and CRC development in the carriers stratified on the basis of the MMR gene germline mutation identified. Table 3 shows the association between risk factors and CRC development in *MLH1* germline mutation carriers. The Hakka ethnicity and manual occupations were associated with an increased CRC risk (HR = 1.64, 95% CI = 1.09–2.48 and HR = 1.63, 95% CI = 1.07–2.47, respectively). However, college education, regular physical activity, ever tea consumption and high-level fruit intake were associated with a decreased CRC risk (HR = 0.56, 95% CI = 0.32–0.99; HR = 0.55, 95% CI = 0.35–0.86; HR = 0.62, 95% CI = 0.42–0.91; and HR = 0.58, 95% CI = 0.35–0.94, respectively).

**Table 3:** Hazard ratios for association between demographic variables, lifestyle factors, or dietary factors and colorectal cancer (CRC) risk in *MLH1* germline mutation carriers.

Variable	Number of carriers	Person-Year	CRC cases	HR (95% CI)^c^	P value
Sex					
Female	107	4,870	61	1.00	
Male	102	4,203	53	1.08 (0.75–1.57)	0.661
Education					
Elementary	74	3,951	58	1.00	
High school	69	2,727	35	0.90 (0.55–1.46)	0.672
College	66	2,396	21	**0.56 (0.32–0.99)**	**0.049**
Ethnicity					
Taiwanese	154	6,748	80	1.00	
Hakka	48	2,042	29	**1.64 (1.09–2.48)**	**0.017**
Other ^a^	7	284	5	1.61 (0.74–3.53)	0.231
Occupation					
Skilled	89	3,825	42	1.00	
Manual	78	3,567	50	**1.63 (1.07–2.47)**	**0.021**
Other ^b^	39	1,523	19	1.83 (0.97–3.44)	0.061
Blood group					
O	81	3,728	47	1.00	
A	73	3,140	45	1.12 (0.74–1.70)	0.584
B	38	1,585	16	0.90 (0.52–1.52)	0.667
AB	8	367	5	1.10 (0.47–2.59)	0.882
Regular physical activity					
No	145	6,251	89	1.00	
Yes	64	2,823	25	**0.55 (0.35–0.86)**	**0.009**
Cigarette smoking					
Never	148	6,435	81	1.00	
Ever	61	2,639	33	0.98 (0.65–1.48)	0.904
Alcohol drinking					
Never	148	6,606	89	1.00	
Ever	61	2,468	25	0.73 (0.45–1.16)	0.185
Tea consumption					
Never	94	4,334	64	1.00	
Ever	115	4,740	50	**0.62 (0.42–0.91)**	**0.013**
Coffee consumption					
Never	142	6,572	88	1.00	
Ever	67	2,502	26	0.83 (0.52–1.32)	0.439
Meat intake					
Low	72	2,823	36	1.00	
Medium	68	3,046	40	1.04 (0.67–1.63)	0.847
High	69	3,204	38	0.95 (0.61–1.49)	0.846
Vegetable intake					
Low	76	3,447	44	1.00	
Medium	69	2,848	38	1.38 (0.87–2.18)	0.171
High	64	2,779	32	0.88 (0.56–1.38)	0.584
Fruit intake					
Low	64	2,638	35	1.00	
Medium	71	3,251	49	1.03 (0.65–1.65)	0.871
High	74	3,185	30	**0.58 (0.35–0.94)**	**0.027**
Seafood intake					
Low	96	4,264	57	1.00	
Medium	83	3,409	41	0.87 (0.57–1.34)	0.554
High	30	1,401	16	0.88 (0.51–1.52)	0.661
Staple food intake					
Low	66	2,659	33	1.00	
Medium	85	3,761	50	0.95 (0.59–1.51)	0.835
High	58	2,654	31	0.69 (0.43–1.11)	0.133

^a^ Mainland Chinese and aborigines

^b^ Retirees and working as housekeepers

^c^ Robust variance estimation for familial correlation between risk factors and CRC risk

Among *MSH2* germline mutation carriers, those with blood group B exhibited an approximately 2.64 times higher CRC risk (95% CI = 1.06–6.58) than did those with blood group O (Table 4). Those who consumed alcohol exhibited an approximately 2.33 times higher CRC risk (95% CI = 1.04–5.21) than did those who never drank alcohol. However, no significant association was observed among the other risk factors and CRC risk.

**Table 4:** Hazard ratios for association between demographic variables, lifestyle factors, or dietary factors and colorectal cancer (CRC) risk in *MSH2* germline mutation carriers.

Variable	Number of carriers	Person-Year	CRC cases	HR (95% CI) ^c^	P value
Sex					
Female	49	1,863	15	1.00	
Male	43	1,591	18	1.03 (0.49–2.01)	0.944
Education					
Elementary	26	1,284	16	1.00	
High school	37	1,385	12	0.63 (0.20–1.98)	0.431
College	29	786	5	2.50 (0.44–14.1)	0.298
Ethnicity					
Taiwanese	81	3,015	27	1.00	
Hakka	11	439	6	2.76 (0.89–8.59)	0.078
Other ^a^	–	–	–	–	–
Occupation					
Skilled	30	1,149	10	1.00	
Manual	34	1,406	19	2.60 (0.88–7.73)	0.084
Other ^b^	28	899	4	0.88 (0.28–2.80)	0.832
Blood group					
O	46	1,802	15	1.00	
A	23	787	5	0.66 (0.17–2.53)	0.523
B	16	688	12	**2.64 (1.06–6.58)**	**0.036**
AB	–	–	–	–	–
Regular physical activity					
No	52	1,896	20	1.00	
Yes	40	1,559	13	0.64 (0.26–1.59)	0.337
Cigarette smoking					
Never	62	2,327	21	1.00	
Ever	30	1,127	12	1.28 (0.58–2.83)	0.544
Alcohol drinking					
Never	54	2,059	16	1.00	
Ever	38	1,396	17	**2.33 (1.04–5.21)**	**0.038**
Tea consumption					
Never	26	1,078	10	1.00	
Ever	66	2,377	23	1.30 (0.48–3.58)	0.606
Coffee consumption					
Never	59	2,272	21	1.00	
Ever	33	1,183	12	1.56 (0.69–3.51)	0.283
Meat intake					
Low	35	1,198	8	1.00	
Medium	34	1,243	13	1.39 (0.51–3.83)	0.514
High	23	1,014	12	1.63 (0.47–5.65)	0.434
Vegetable intake					
Low	33	1,219	11	1.00	
Medium	32	1,250	11	0.59 (0.19–1.82)	0.359
High	27	985	11	1.06 (0.47–2.36)	0.877
Fruit intake					
Low	47	1,870	21	1.00	
Medium	20	817	7	0.63 (0.27–1.44)	0.281
High	25	768	5	1.22 (0.34–4.33)	0.751
Seafood intake					
Low	47	1,810	18	1.00	
Medium	32	1,166	9	0.60 (0.26–1.38)	0.233
High	13	478	6	1.31 (0.46–3.76)	0.607
Staple food intake					
Low	36	1,245	8	1.00	
Medium	29	1,218	15	2.14 (0.81–5.63)	0.121
High	27	992	10	2.13 (0.68–6.65)	0.191

^a^ Mainland Chinese and aborigines

^b^ Retirees and working as housekeepers

^c^ Robust variance estimation for familial correlation between risk factors and CRC risk

Table 5 lists the results of the multivariate analysis of the association between the risk factors and CRC development. Among the total cohort, subjects of the Hakka ethnicity exhibited a significantly increased CRC risk (HR = 1.62, 95% CI = 1.09–2.34); however, those who performed regular physical activity exhibited a significantly decreased CRC risk (HR = 0.62, 95% CI = 0.41–0.88). These significant risks were pronounced in *MLH1* germline mutation carriers, with corresponding HRs of 1.72 (95% CI = 1.16–2.55) and 0.54 (95% CI = 0.34–0.83) among subjects of the Hakka ethnicity and those who performed regular physical activity, respectively. In addition, the total cohort with a manual occupation had a 1.56 times higher CRC risk (95% CI = 1.07–2.27) than did that with a skilled occupation. After stepwise selection model in *MSH2* germline mutation carriers, only blood group remain significant and its results are the same as in Table 4.

**Table 5:** Multivariate Cox proportional hazard model for CRC risk in carriers with germline mutations in mismatch repair genes.

Variable	Total cohort		*MLH1* germline mutation carriers	
	HR (95% CI) ^c^	P value	HR (95% CI) ^d^	P value
Ethnicity				
Taiwanese	1.00		1.00	
Hakka	**1.62 (1.09–2.34)**	**0.015**	**1.72 (1.16–2.55)**	**0.006**
Other ^a^	1.56 (0.67–3.64)	0.301	1.57 (0.64–3.83)	0.321
Occupation				
Skilled	1.00		–	
Manual	**1.56 (1.07–2.27)**	**0.021**		
Other ^b^	1.19 (0.67–2.11)	0.551		
Regular physical activity				
No	1.00		1.00	
Yes	**0.62 (0.41–0.88)**	**0.009**	**0.54 (0.34–0.83)**	**0.005**

^a^ Mainland Chinese and aborigines

^b^ Retirees and working as housekeepers

^c^ Robust variance estimation for familial correlation between risk factors and CRC risk and further adjusted for the specific MMR genes mutated in carriers

^d^ Robust variance estimation for familial correlation between risk factors and CRC risk

We further analysed data without probands, using a multivariate Cox proportional hazard model, which revealed that Hakka ethnicity was associated with an increased CRC risk in both the total cohort (HR = 2.23, 95% CI = 1.36–3.64) and *MLH1* germline mutation carriers (HR = 2.50, 95% CI = 1.42–4.39) (S2 Table). *MSH2* germline mutation carriers with blood group type B had a 3.5-times higher risk of CRC (95% CI = 1.11–10.9) than those with blood group type O. However, regular physical activity was associated with a decreased CRC risk for *MLH1* germline mutation carriers (HR = 0.52, 95% CI = 0.29–0.92). Although manual occupation was not significantly associated with CRC risk, it also exhibited a positive association.

## Discussion

This study revealed that Hakka ethnicity, manual occupation, and blood group type B were associated with an increased CRC risk, whereas regular physical activity was associated with a decreased CRC risk. The risk factors thought to be associated with CRC in patients with Lynch syndrome varied between people with *MLH1* and *MSH2* germline mutations. In *MLH1* mutation carriers, CRC risk was associated with Hakka ethnicity and regular physical activity, whereas in *MSH2* mutation carriers, it was associated with blood group type B.

The multivariate model revealed that Hakka ethnicity was associated with a higher CRC risk in the total cohort (HR = 1.62, 95% CI = 1.09–2.34) and *MLH1* germline mutation carriers (HR = 1.72, 95% CI = 1.16–2.55) than in their Taiwanese counterparts. No study has investigated this association; thus, the mechanism underlying the increased risk of CRC development in this ethnic group remains unclear. However, Pan et al. investigated the associations of genetic polymorphisms in glutathione S-transferase genes of the Hakka population of south China with family histories of certain chronic diseases and reported that the Hakka population exhibits distinct genetic structures, possibly because of factors related to long-distance migration that occurred centuries ago [24].

Manual occupation was significantly associated with an increased risk of CRC development (HR = 1.56, 95% CI = 1.07–2.27) in the total cohort compared with skilled occupation. Workers with a manual occupation (blue collar) were from farming, architectural, and labor-related industries. These industries have been previously reported to expose workers to harmful chemicals [25]. Manual workers in these industries are exposed to more potential hazardous chemicals and physical factors than skilled workers (white collar) in ambient environment or those in other nonoccupational settings such as retirees [26].

The association between blood group type and CRC risk has rarely been investigated. To date, only one case-control study has reported an association between blood group type O and CRC risk [27]. However, our study revealed that only *MSH2* germline mutation carriers with blood group B exhibited an increased CRC risk (HR = 2.64, 95% CI = 1.06–6.58). Moreover, a study reported that people with blood group type B exhibit an increased risk of cancer development; which is consistent with our findings, although the primary outcome of that study was the development of pancreatic cancer and not CRC [28].

The association between physical activity and CRC in Lynch syndrome patients has rarely been investigated because most studies focus on sporadic CRC cases. In this study, we found a risk reduction of 38% in the total cohort and 46% in the *MLH1* germline mutation carriers, which is consistent with the results of two previous sporadic CRC studies [15,19]. Yeh et al. reported a risk reduction of approximately 50% in men performing vigorous leisure time activities [15]. Song et al. reported that increased physical activity reduces the prevalence of colorectal adenoma by 44% in Koreans [19]. The protective effect of regular physical activity against CRC has been suggested previously by several researchers. Sanchez et al. and Simons et al. reported that physical activity may reduce weight [16] and improve immune system and insulin sensitivity [29], both of which have been associated with a decreased CRC risk. Another study suggested that regular physical activity may increase peristaltic movements, thereby reducing the duration of mucosal exposure to carcinogens [30].

A major strength of our study was that all study participants were confirmed to have hereditary MMR gene germline mutations. A structured baseline questionnaire enabled us to explore several risk factors, which enabled us to generalize our findings to people with *MLH1* and *MSH2* germline mutations in clinical settings. However, a major concern is potential ascertainment bias. The Amsterdam II criteria were used for patient ascertainment, hence some families with MMR germline mutations were missed. Syngal et al. reported sensitivity of 78% and specificity of 61% for the Amsterdam II criteria. However, the Amsterdam II criteria are a better screening tool than Amsterdam or modified Amsterdam criteria [31]. The ascertainment bias is minimal as the same risk factors (Hakka ethnicity, blood group type B and regular physical activity) of CRC development were observed from our multivariate Cox regression models for data analyzed with and without probands. In addition, this study was limited by our inability to test for other MMR gene germline mutations that are also associated with Lynch syndrome, such as *MSH6*, *PMS2* and *EPCAM* germline mutations. We found two patients with mutations in both *MLH1* and *MSH2*. Because the risk of CRC development could be higher in patients with two different mutations than in those with one mutation, these two patients were excluded from this study. Further study with more subjects with multiple MMR gene germline mutations is required to explore this relationship. In addition, approximately 54% of the mutation carriers were not willing to be followed up for their recent morbidity status at least once since recruitment; hence, we likely failed to record some newly developed cancer cases. However, if the identified risk factors are associated with CRC risk in *MLH1* or *MSH2* germline mutation carriers, this misclassification would bias the results toward the null hypothesis and lead to underestimation of the risk.

In conclusion, this study revealed that Hakka ethnicity, manual occupation, and blood group type B were associated with an increased CRC risk, whereas regular physical activity was associated with a decreased CRC risk. We therefore suggest that all Lynch syndrome patients must be encouraged to adjust their lifestyle by engaging in regular physical activity. Moreover, regular colonoscopy surveillance should be encouraged among these patients, particularly among those of Hakka ethnicity or with blood group type B.

## Supporting Information

S1 Table Germline *MLH1* and *MSH2* mutations in Taiwanese families with Lynch syndrome. (DOCX)

S2 Table Multivariate Cox proportional hazard model for CRC risk in family members with germline mutations in mismatch repair genes. (DOCX)
