# Supplementary material for: Risk Factors Associated with Colorectal Cancer in a Subset of Patients with Mutations in MLH1 and MSH2 in Taiwan Fulfilling the Amsterdam II Criteria for Lynch Syndrome
Source: PLoS One. 2015 Jun 8;10(6):e0130018. doi: 10.1371/journal.pone.0130018 (PMC4460082; doi:10.1371/journal.pone.0130018)
Supplement: S1 Table — (DOCX) [file pone.0130018.s001.docx]

**S1 Table. Germline *MLH1* and *MSH2* mutations in Taiwanese families with Lynch syndrome.**

| **Event** | **Gene** | **Exon /Intron** | **Nucleotide** | **Codon** | **Consequence** | **Family ID** | **Having been Reported^a^** |
| --- | --- | --- | --- | --- | --- | --- | --- |
| 1 | *MLH1* | 1 | c.105_106insAA | 35 | Frameshift | A057, J061, A121 | Yes |
| 2 | *MLH1* | 2 | c.122AT>TA | 41 | Asp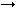Val | A017 | No |
| 3 | *MLH1* | 3 | c.229T>C | 77 | Cys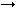Arg | A075 | Yes |
| 4 | *MLH1* | 3 | c.250A>G | 84 | Lys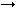Glu | A048 | Yes |
| 5 | *MLH1* | 3 | c.298C>T | 100 | Arg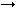Stop | A012 | Yes |
| 6 | *MLH1* | 4 | c.320T>G | 107 | Ile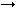Arg | A119 | Yes |
| 7 | *MLH1* | 4 | c.350C>T | 117 | Thr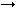Met | O102, A034, A127 | Yes |
| 8 | *MLH1* | 6 | c.472delA | 158 | Frameshift | A079 | No |
| 9 | *MLH1* | 10 | c.793C>T | 265 | Arg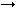Cys | Founder^b^ | Yes |
| 10 | *MLH1* | 11 | c.1038G>T | 346 | Gln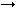His | A088 | No |
| 11 | *MLH1* | 12 | c.1178T>C | 393 | Leu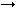Pro | A056 | No |
| 12 | *MLH1* | 12 | c.1349delA | 450 | Frameshift | K076 | No |
| 13 | *MLH1* | 13 | c. 1489dupC | 495-497 | Frameshift | A124, A151 | Yes |
| 14 | *MLH1* | Intron 13 | c.1558+5G>A |  | Splice defect | A107 | No |
| 15 | *MLH1* | 15 | c.1731G>A | 577 | Aberrant splicing | A039 | Yes |
| 16 | *MLH1* | 16 | c.1761_1762insG | 587 | Frameshift | J059 | No |
| 17 | *MLH1* | 16 | c.1771delG | 591 | Frameshift | A040 | No |
| 18 | *MLH1* | 16 | c.1846_1848delAAG | 616 | del Lys | 6 families[^c^](http://onlinelibrary.wiley.com/doi/10.1111/j.1399-0004.2009.01162.x/full#t1n4) | Yes |
| 19 | *MLH1* | 17 | c.1989G>A | 663 | Aberrant splicing | A031 | Yes |
| 20 | *MLH1* | 19 | c.2172_2173insG | 724 | Frameshift | A054 | No |
| 21 | *MLH1* | 19 | c.2263A>G | 755 | Arg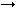Gly | K058, J080 | No |
| 22 | *MLH1* |  | deletion exon 5 |  | Large deletion | A098 | No |
| 23 | *MLH1* |  | deletion exons 11–15 |  | Large deletion | 4 families^d^ | No |
| 24 | *MLH1* |  | deletion exons 13 |  | Large deletion | A096 | Yes |
| 25 | *MLH1* |  | deletion exons 16–19 |  | Large deletion | A068 | No |
| 26 | *MSH2* | 1 | c.142C>T | 48 | Glu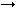Stop | A085 | No |
| 27 | *MSH2* | Intron 5 | c.942 +3A>T |  | Splice defect | O105 | Yes |
| 28 | *MSH2* | 6 | c.1035G>A | 345 | Trp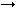Stop | O095 | Yes |
| 29 | *MSH2* | 7 | c.1226_1227delAG | 409 | Frameshift | O090, A144 | Yes |
| 30 | *MSH2* | Intron 7 | c.1277-2A>C |  | Splice defect | A122 | Yes |
| 31 | *MSH2* | 9 | c.1440_1441insA | 480 | Frameshift | A003, A009 | No |
| 32 | *MSH2* | Intron 10 | c.1661+1G>A |  | Splice defect | A136 | Yes |
| 33 | *MSH2* | 12 | c.1835C>G | 612 | Ser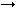Stop | A049 | Yes |
| 34 | *MSH2* | 12 | c.1886A>G | 629 | Gln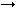Arg | O095 | Yes |
| 35 | *MSH2* | 13 | c.2087C>T | 696 | Pro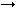Leu | A047, A092 | No |
| 36 | *MSH2* | 13 | c.2131C>T | 711 | Arg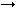Stop | K086 | Yes |
| 37 | *MSH2* | 15 | c.2482_2483insA | 828 | Frameshift | J060 | No |
| 38 | *MSH2* | 15 | c.2516A>G | 839 | His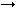Arg | O073[^e^](http://onlinelibrary.wiley.com/doi/10.1111/j.1399-0004.2009.01162.x/full#t1n6), A010 | Yes |
| 39 | *MSH2* | 15 | c.2595_2597delCAT | 865-866 | del Ile | A156[^e^](http://onlinelibrary.wiley.com/doi/10.1111/j.1399-0004.2009.01162.x/full#t1n6) | No |
| 40 | *MSH2* |  | deletion exon 1 |  | Large deletion | A097, A132 | Yes |
| 41 | *MSH2* |  | deletion exons 1–6 |  | Large deletion | A019 | Yes |
| 42 | *MSH2* |  | deletion exons 1–7 |  | Large deletion | A115 | Yes |
| 43 | *MSH2* |  | deletion exon 4 |  | Large deletion | A062, A122 | Yes |
| 44 | *MSH2* |  | deletion exon 7 |  | Large deletion | A041 | Yes |
| 45 | *MSH2* |  | deletion exon 8 |  | Large deletion | A013 | Yes |
| 46 | *MSH2* |  | deletion exons 9–10 |  | Large deletion | A087 | Yes |
| 47 | *MSH2* |  | deletion exon 11 |  | Large deletion | A099 | Yes |

^a^ According to InSiGHT database (http://www.insight-group.org/), MMR Genes Variant Database (http://www.med.mun.ca/MMRvariants/), and Human Gene Mutation Database (http://www.hgmd.cf.ac.uk/ac/index.php).

^b^ Including 18 families (A004, A007, A008, A024, A036, A042, A045, A046, A072, O073, A077, A100, A104, A131, A134, A140, M146, and A149).

^c^ Including 6 families (A015, J083, O091, J103, A106, and A156).

^d^ Including 4 families (A029, A081, A094, and A101).

^e^ Families harboring double mutations (O073 and A156).
