# Supplementary material for: Risk Factors Associated with Colorectal Cancer in a Subset of Patients with Mutations in MLH1 and MSH2 in Taiwan Fulfilling the Amsterdam II Criteria for Lynch Syndrome
Source: PLoS One. 2015 Jun 8;10(6):e0130018. doi: 10.1371/journal.pone.0130018 (PMC4460082; doi:10.1371/journal.pone.0130018)
Supplement: S2 Table — (DOCX) [file pone.0130018.s002.docx]

**S2 Table. Multivariate Cox proportional hazard model for CRC risk in family members with germline mutations in mismatch repair genes.**

| Variable | Total cohort  HR (95% CI) ^c^ | P value | *MLH1* germline  mutation carriers  HR (95% CI) ^d^ | P value | *MSH2* germline  mutation carriers  HR (95% CI) ^d^ | P value |
| --- | --- | --- | --- | --- | --- | --- |
| Ethnicity |  |  |  |  |  |  |
| Taiwanese | 1.00 |  | 1.00 |  | – |  |
| Hakka | **2.23 (1.36–3.64)** | **0.001** | **2.50 (1.42–4.39)** | **0.001** |  |  |
| Other ^a^ | 1.42 (0.13–15.1) | 0.774 | 1.44 (0.11–17.5) | 0.773 |  |  |
| Occupation |  |  |  |  |  |  |
| Skilled | 1.00 |  | – |  | – |  |
| Manual | 1.37 (0.84–2.24) | 0.210 |  |  |  |  |
| Other ^b^ | 0.93 (0.43–2.02) | 0.862 |  |  |  |  |
| Blood group |  |  |  |  |  |  |
| O | – |  | – |  | 1.00 |  |
| A |  |  |  |  | 0.79 (0.15–3.96) | 0.773 |
| B |  |  |  |  | **3.50 (1.11–10.9)** | **0.032** |
| AB |  |  |  |  | – | – |
| Regular physical  activity |  |  |  |  |  |  |
| No | 1.00 |  | 1.00 |  | – |  |
| Yes | 0.61 (0.35–1.03) | 0.068 | **0.52 (0.29–0.92)** | **0.024** |  |  |

^a^ Mainland Chinese and aborigines

^b^ Retirees and working as housekeepers

^c^ Robust variance estimation for familial correlation between risk factors and CRC risk and further adjusted for the specific MMR genes mutated in carriers

^d^ Robust variance estimation for familial correlation between risk factors and CRC risk
